# Sociodemographic risk factors for coronavirus disease 2019 (COVID-19) infection among Massachusetts healthcare workers: A retrospective cohort study

**DOI:** 10.1017/ice.2021.17

**Published:** 2021-01-28

**Authors:** Fan-Yun Lan, Robert Filler, Soni Mathew, Jane Buley, Eirini Iliaki, Lou Ann Bruno-Murtha, Rebecca Osgood, Costas A. Christophi, Alejandro Fernandez-Montero, Stefanos N. Kales

**Affiliations:** 1 Department of Environmental Health, Harvard University T.H. Chan School of Public Health, Boston, Massachusetts, United States; 2 Department of Occupational and Environmental Medicine, National Cheng Kung University Hospital, College of Medicine, National Cheng Kung University, Tainan, Taiwan; 3 Division of Occupational Medicine, Cambridge Health Alliance, Harvard Medical School, Cambridge, Massachusetts, United States; 4 Division of Infection Prevention and Infectious Diseases,Cambridge Health Alliance, Harvard Medical School, Cambridge, Massachusetts, United States; 5 Division of Pathology, Cambridge Health Alliance, Harvard Medical School, Cambridge, Massachusetts, United States; 6 Pathology Department, Massachusetts General Hospital, Harvard Medical School, Boston, Massachusetts, United States; 7 Cyprus International Institute for Environmental and Public Health, Cyprus University of Technology, Limassol, Cyprus; 8 Department of Occupational Medicine, University of Navarra, Pamplona, Spain

## Abstract

**Objective:**

To better understand coronavirus disease 2019 (COVID-19) transmission among healthcare workers (HCWs), we investigated occupational and nonoccupational risk factors associated with cumulative COVID-19 incidence among a Massachusetts HCW cohort.

**Design, setting, and participants:**

The retrospective cohort study included adult HCWs in a single healthcare system from March 9 to June 3, 2020.

**Methods:**

The SARS-CoV-2 nasopharyngeal RT-PCR results and demographics of the study participants were deidentified and extracted from an established occupational health, COVID-19 database at the healthcare system. HCWs from each particular job grouping had been categorized into frontline or nonfrontline workers. Incidence rate ratios (IRRs) and odds ratios (ORs) were used to compare subgroups after excluding HCWs involved in early infection clusters before universal masking began. A sensitivity analysis was performed comparing jobs with the greatest potential occupational risks with others.

**Results:**

Of 5,177 HCWs, 152 (2.94%) were diagnosed with COVID-19. Affected HCWs resided in areas with higher community attack rates (median, 1,755.2 vs 1,412.4 cases per 100,000; *P* < .001; multivariate-adjusted IRR, 1.89; 95% CI, 1.03–3.44 comparing fifth to first quintile of community rates). After multivariate adjustment, African-American and Hispanic HCWs had higher incidence of COVID-19 than non-Hispanic white HCWs (IRR, 2.78; 95% CI, 1.78–4.33; and IRR, 2.41, 95% CI, 1.42–4.07, respectively). After adjusting for race and residential rates, frontline HCWs had a higher IRR (1.73, 95% CI, 1.16–2.54) than nonfrontline HCWs overall, but not within specific job categories nor when comparing the highest risk jobs to others.

**Conclusions:**

After universal masking was instituted, the strongest risk factors associated with HCW COVID-19 infection were residential community infection rate and race.

The health of healthcare workers (HCWs) during the coronavirus disease 2019 (COVID-19) pandemic is of great public interest.^1^ Many HCWs are essential workers, unprotected by stay-at-home mitigation measures.^2,3^ Adequate infection prevention measures for HCWs are critical because of the high risk of coming into contact with COVID-19 patients as well as potentially contaminated environments.^1,4,5^

The occupational risk factors of COVID-19 infection for HCWs include inadequate use of personal protective equipment (PPE), close contact with source patients and infected coworkers, suboptimal hand hygiene, and long working hours.^6-8^ Although contact with COVID-19 cases increases the risk of being infected, no robust evidence has demonstrated higher risk in frontline HCWs versus nonfrontline workers.^9^ One of the main reasons for this is the protective effect of PPE.^10^ Epidemiological research indicates that proper PPE provides good protection against viral transmission to frontline HCWs. A study conducted in China showed that none of the HCWs deployed to Wuhan during the outbreak tested positive after 6–8 weeks of serving on the front line.^11^

Although potential infection transmission routes in HCW work environments have been established and evidence-based infection control strategies have been implemented in healthcare facilities accordingly,^12^ little research has investigated nonoccupational risk factors for HCWs. Race-related health disparities have been reported in the COVID-19 pandemic, with minorities being at higher risk of morbidity and mortality.^13^ In addition, residential community infection rates may contribute to HCW COVID-19 incidence because most HCWs have continued to have personal exposures with their families and communities. A recently published Dutch study investigated the severe acute respiratory coronavirus virus 2 (SARS-CoV-2) virology among HCWs and hospitalized patients and found no evidence of nosocomial infection.^14^ The study indirectly supports the hypothesis that COVID-19 infection among HCWs may be driven by community attack rates or other nonoccupational factors. Therefore, we conducted this study to examine HCW occupational risks with regards to their working departments and jobs, as well as their nonoccupational risks, to identify the main contributors to COVID-19 incidence among HCWs during the pandemic.

## Methods

### Study population and setting

The HCWs of a Massachusetts community healthcare system have been under surveillance for COVID-19 infection, including from March 9 to the end of the current study period, June 3, 2020. A COVID-19 “hotline” was set up by the occupational medicine service of the healthcare system to telephonically triage any HCW with regard to their symptoms, travel, and exposure history, followed by further referral for SARS-CoV-2 testing as clinically indicated. To optimally understand HCW infection rates, the occupational health hotline also collected HCW demographic and administrative information, which included age, race, sex, and their residential area from the system’s human resources department.

All HCWs >18 years of age and actively serving in the healthcare system by June 3, 2020, were retrospectively included in this study. The contracted, permanently remote work force consisting of HCWs residing outside the New England area was excluded for the analyses involving residential locations.

The details of the HCW cohort, triage, and testing have been published previously.^15^

### Outcome measurement

The details of the outcome measurement are available in the published study,^15^ and are only summarized here. The outcome of interest was COVID-19 as confirmed by SARS-CoV-2 testing results and the corresponding cumulative incidence of COVID-19 as of June 3, 2020. After an HCW contacted the hotline due to either (1) travel; (2) potential contact with a COVID-19–positive/–suspected person; or (3) possible viral symptoms and was telephonically interviewed, the HCW was referred for a nasopharyngeal swab. Specimens were transferred to a laboratory for a real-time, reverse-transcriptase–polymerase-chain-reaction (RT-PCR) diagnostic panel that qualitatively detected nucleic acid from SARS-CoV-2. The test has limits of detection ranging from 600 to 5,400 nucleic acid-based amplification tests (NAATs) detectable units/mL.^16^ For those with initial negative assays but progressive symptom(s), a second testing referral was arranged after the hotline follow-up telephonic interviews.

We considered HCWs as clinically COVID-19 free if they never underwent a hotline triage; were never tested, or consistently tested negative for SARS-CoV-2. We considered HCWs as COVID-19 cases if they had any positive PCR assays during the study period. Each HCW was only entered once into the final database used for the analyses.

### Risk factors of interest

From human resources, we collected data on HCWs’ working locations, job titles, and whether they worked at the front line or faced patients directly. The research team held meetings with human resources and infection prevention leadership of the healthcare system to best classify the occupational status of each HCW during the pandemic. We defined a total of 15 job groups: registered nurse (RN), other nurse (ie, licensed practical nurse, medical assistant, nursing assistant), medical provider (ie, medical doctor, doctor of osteopathic medicine, physician assistant, nurse practitioner, resident, and midwife), clinical support (including practice manager, practice medical receptionist, etc), mental health provider/social worker/psychologist, milieu counselor/social worker on in-patient psychiatry unit, rehabilitation, ancillary job (ie, security, food service, facility), radiology technician, pharmacy, laboratory (nonmedical doctor), interpreter, dental hygiene/dental assistant, administrative worker (including human resource, finance, information technician, nonmedical administrator, and medical records), and others. All 15 job groups were further categorized into either frontline or nonfrontline based on whether the workers had been assigned to shifts at the COVID-19 testing sites, at the COVID-19 units, or in COVID-19 clinics and whether the HCW did patient-facing or non–patient-facing work for the job groups that were ineligible for the frontline work assignments (eg, radiology department), Notably, dental hygienist and dental assistants were all patient-facing, and administrative workers were all nonfrontline.

Nonoccupational variables, including demographic information and residential area, were cross validated between the medical personnel of the occupational service hotline and the human resource department of the healthcare system. We obtained the cumulative COVID-19 attack rate in the residential area of each HCW to the city or town level (Massachusetts)^17^ or the county level (other states in the New England area)^18^ based on the residential zip code in the database.

### Data collection and ethical statement

All relevant data were extracted from the hotline database up to June 3, 2020. The database was electronically stored on a secured sever and was accessible only through password-protected computers. The database was deidentified prior to statistical analysis. The research protocol was reviewed by the institutional review board of the healthcare system and was determined to be exempt from human-subject research approval based on the use of existing, HIPAA-deidentified data.^15^

### Cluster infection

We identified the HCW cases that were involved in workplace clusters that were likely to be HCW-to-HCW transmission and analyzed them separately. The clusters all took place in the earliest stage of the pandemic (ie, prior to mid April) and started before universal masking^19^ was implemented in an effort to conserve initially limited mask supplies. Therefore, because universal masking intervention could have fundamentally changed the dynamics around transmission, we excluded such cases from the main analysis.

### SARS-CoV-2 testing propensity

Since SARS-CoV-2 testing was prioritized for HCWs,^20,21^ the overall testing propensity was higher among the study population than the general population of Massachusetts during the study period. We calculated the testing rate of the healthcare system and had the rate divided by the community testing rate^17^ to derive a testing propensity weight. This weight was taken into account when comparing the cumulative COVID-19 attack rate among the HCWs and their respective communities of residence.

### Statistical analyses

Categoric variables were reported as counts with percentages, and the data were compared between groups using the χ^2^ test of independence after the Yates continuity correction or the Fisher exact test, as appropriate. Continuous variables were checked for normality with Q-Q plots, and are reported as mean ± standard deviation or median (Q1–Q3), as appropriate, and the data were compared between groups with the parametric *t* test or the nonparametric Wilcoxon test, respectively. For HCW residential community cumulative attack rates, we calculated the median and 95% confidence interval (CI) using exact methods or bootstrapping methods if exact methods were not applicable. We further categorized the residential community cumulative attack rates into quintiles, and we demonstrated the case distribution trends across the quintiles. No imputation was made for missing data.

We used Poisson and logistic regression models to calculate incidence rate ratios (IRRs) or odds ratios (ORs), regressing PCR-diagnosed COVID-19 positivity on the sociodemographic factors of interest (ie, age, sex, race, residential area COVID-19 cumulative attack rate, and frontline working status). The variable selection was based on our domain knowledge and existing evidence.^13,14^ Point estimates and 95% CIs are reported.

All *P* values presented are 2-tailed. A *P* < .05 was considered statistically significant. R version 3.6.3 statistical software (R Foundation for Statistical Computing, Vienna, Austria) was used for the analyses.

### Sensitivity analysis

We analyzed HCWs deemed by infection prevention as having the greatest presumed potential risks of exposure in a sensitivity analysis for occupational risks. Those HCWs included SARS-CoV-2 testing personnel, anesthesia specialists, intensive care unit (ICU) providers, ICU RNs, other ICU nurses, respiratory technicians, emergency room (ER) providers, ER RNs, and other ER nurses. IRRs were calculated to compare the HCWs of the greatest presumed risk group versus other HCWs.

## Results

In total, 5,177 adult HCWs were actively working in the healthcare system as of June 3, 2020. They were all included in the initial analyses. Among them, 152 (2.94%) were clinically diagnosed with COVID-19 after laboratory confirmed SARS-CoV-2 positive PCR assays, and another 5,025 HCWs were either clinically COVID-19 free without SARS-CoV-2 testing referral (n = 4,276) or had consistently negative PCR assays (n = 749). Among the 901 occupational hotline-triaged HCWs, 14 (1.6%) had a travel history, 225 (25.0%) had potential contact with a COVID-19–positive/–suspected person, and 812 (90.1%) had possible viral symptoms. Also, 240 HCWs (26.6%) received >1 PCR testing referral. For the entire cohort, the average age was 44.3 ± 13.5 years old, and most were female (75.0%). There was no age or sex difference between positive cases and negative subjects, but COVID-19 cases were more likely to be nonwhite minorities (African-American 36.8% vs 16.5%; Hispanic 19.7% vs 11.2%; *P* < .001). In addition, COVID-19 cases lived in communities with significantly higher average cumulative attack rates (median, 1,755.2 vs 1,412.4 cases per 100,000; *P* < .001) (Table 1).

**Table 1.** Demographic Characteristics of the Health System’s Workforce, Overall and by Clinical COVID-19 Diagnosis

Variable	Overall (N = 5177)	Clinical COVID-19 (N = 152)	Negative Testing or Clinically COVID-19 Free (N = 5025)	*P* Value
Age (n = 5,177), mean y ±SD	44.3±13.5	42.7±12.7	44.3±13.5	.132
**Sex (n = 4,879), no (%)**				
Female	3,657 (75.0)	107 (70.4)	3,550 (70.6)	.818
Male	1,222 (25.0)	38 (25.0)	1,184 (23.6)	
**Race (n = 4556), no (%)**				
Non–Hispanic white	2,556 (56.1)	50 (32.9)	2,506 (49.9)	**<.001**
African American	887 (19.5)	56 (36.8)	831 (16.5)	
Hispanic	594 (13.0)	30 (19.7)	564 (11.2)	
Others	519 (11.4)	8 (5.3)	511 (10.2)	
Residential area COVID-19 cumulative attack rate (per 100,000)^a^	1,521.8 (907.9–2,014.8) (n = 4,600)	1,755.2 (1,348.3–3,393.3) (n = 145)	1,412.4 (889.3–1,930.6) (n = 4,455)	**<.001** ^b^

a Limited to those residing in New England area. Median (Q1–Q3) for residential area COVID-19 cumulative attack rate.

b Wilcoxon rank sum test with continuity correction.

During the research period, the IRR for African-American HCWs was 3.37 (95% CI, 2.18–5.20) and that for Hispanic HCWs was 3.17 (95% CI, 1.94–5.16) compared to non-Hispanic white HCWs. After adjusting for age, sex, residential community rates, and frontline or patient-facing working status, the IRRs for African-American and Hispanic HCWs were 2.78 (95% CI, 1.78–4.33) and 2.41 (95% CI, 1.42–4.07), respectively, compared to the COVID-19 incidence among non-Hispanic white HCWs (Table 2).

**Table 2.** Cumulative Clinical COVID-19 Attack Rates for Health System Employees by Sex, Race, and Quintiles of Workers’ Residential Community Cumulative Attack Rates, Excluding 29 Nonindex Employees Involved in Workplace Infection Clusters

Variable	Crude Rate	Rate per 100,000	Incident Rate Ratio (95% CI)	Median Community Rate per 100,000 (95% CI)^a^	Adjusted Incident Rate Ratio
**Sex (n = 4,850)**					
Female	91/3641	2,499.31	1.21 (0.78–1.88)	1,627.51 (1,412.36–1,634.48)	1.15 (0.74–1.80)
Male	25/1209	2,067.83	1.0 Ref	1,390.02 (1,325.23–1,550.49)	1.0 Ref ^b^
**Race (n = 4,528)**					
Non–Hispanic white	38/2544	1,493.71	1.0 Ref	1,181.65 (1,152.36–1,218.63)	1.0 Ref ^c^
African American	44/875	5,028.57	**3.37 (2.18–5.20)**	1,698.80 (1,698.80–1,755.19)	**2.78 (1.78–4.33)**
Hispanic	28/592	4,729.73	**3.17 (1.94–5.16)**	1,924.57 (1,850.46–1,930.59)	**2.41 (1.42–4.07)**
Other	6/517	1,160.54	0.78 (0.33–1.84)	1,390.02 (1,325.23–1,634.48)	0.70 (0.29–1.66)
**Quintiles of employee residential community cumulative attack rates (n = 4,571)^a^**					
First quintile	17/1115	1,524.66	1.0 Ref	826.36 (826.36–826.36)	1.0 Ref ^d^
Second quintile	7/731	957.59	0.63 (0.26–1.51)	1,112.81 (1,107.21–1,152.36)	0.59 (0.24–1.42)
Third quintile	31/1,089	2,846.65	**1.87 (1.03–3.37)**	1634.48 (1,634.48–1,634.48)	1.46 (0.80–2.66)
Fourth quintile	24/895	2,681.56	1.76 (0.94–3.27)	1,930.59 (1,930.59–1,930.59)	1.34 (0.71–2.52)
Fifth quintile	37/741	4,993.25	**3.27 (1.84–5.82)**	3,393.33 (3,393.33–3,393.33)	**1.89 (1.03–3.44)**

Note. CI, confidence interval.

a Limited to those residing in the New England area.

b Adjusted for age, race, residential area COVID-19 cumulative attack rate, and frontline or patient-facing working status.

c Adjusted for age, sex, residential area COVID-19 cumulative attack rate, and frontline or patient-facing working status.

d Adjusted for age, sex, race, and frontline/patient-facing working status.

**Fig. 1.**
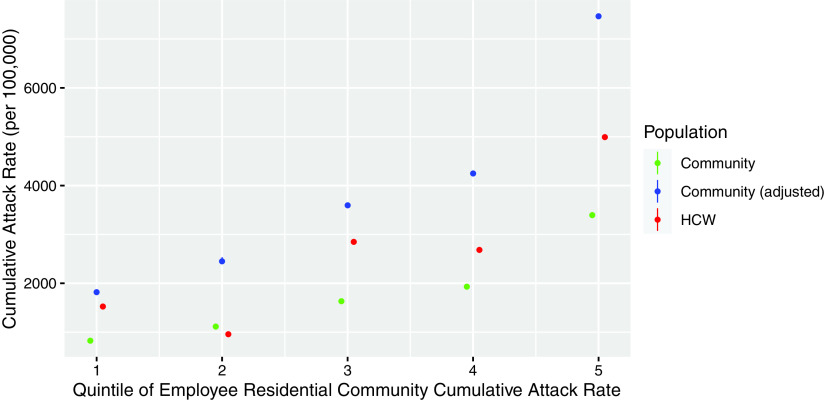
COVID-19 incidence distribution among employees in the health system by quintiles of the residential community cumulative attack rates. Adjusted community rate is the quintile median multiplied by 2.2 to account for higher testing propensity in healthcare workers (HCWs) than the general population of Massachusetts.

COVID-19 cases generally increased across the residential community rate quintile categories of infection rates. Compared to the incidence of HCWs residing in the communities in the lowest quintile cumulative attack rate, the HCWs residing in communities in the second to fifth quintile had the following IRRs: 0.63 for quintile 2, 1.87 for quintile 3, 1.76 for quintile 4, and 3.27 for quintile 5. This incremental effect in IRRs remained even after adjusting for age, sex, race, and frontline/patient-facing working status (adjusted IRR: 0.59, 1.46, 1.34, and 1.89, respectively, across the residential community rate quintile categories of infection rates) (Table 2). We further considered the testing propensity weight, which was 2.2, and the distribution of the cases residing across the quintile categories was largely located between the raw community rate and the adjusted (weighted) one (Fig. 1).

After excluding the 29 non–index-cluster cases, overall frontline or patient-facing HCWs had a 1.73 times odds for being diagnosed with COVID-19 (95% CI, 1.16–2.54) compared to nonfrontline HCWs. Subgroup analyses based on frontline working status were performed for each job category. None of the frontline or patient-facing HCWs had significantly higher odds for being a COVID-19 case, compared to nonfrontline HCWs, after adjusting for race and residential area’s cumulative attack rate (Supplementary Table 1 online). In a further sensitivity analysis, there were 416 HCWs with the greatest presumed exposure (ie, SARS-CoV-2 testing personnel, anesthesia specialists, ICU providers, ICU RNs, other ICU nurses, respiratory technicians, ER providers, ER RNs, and other ER nurses) and 15 (3.6%) of them were diagnosed with COVID-19. When comparing these HCWs of interest with other HCWs (101 of 4,155, 2.4%), the race and community rate-adjusted IRR remained nonsignificant (1.58; 95% CI, 0.92–2.73).

## Discussion

Our study results show that African-American and Hispanic HCWs had higher cumulative COVID-19 incidence rates than non-Hispanic white HCWs. We also found that higher residential community rates were associated with higher COVID-19 incidences among the HCWs. After adjusting for age, sex, frontline or patient-facing working status, and race or community rate, each remained independently associated with higher COVID-19 infection rates. In terms of occupational risks, after excluding clusters that occurred prior to universal masking, we found only modest evidence and inconsistent evidence (within job categories) of higher infection rates among frontline HCWs compared to nonfrontline HCWs for each stratified job group. We detected no significant difference when comparing HCWs of the greatest presumed exposure risk categories with others.

Even though the health of HCWs has been widely discussed during the pandemic, no strong evidence has demonstrated whether HCWs have a higher risk of being infected.^9,11,22,23^ Despite accumulating literature arguing that HCW work-related transmission is not negligible,^2,24^ a recently published study in which whole viral genome sequencing was conducted among infected HCWs in the southern Netherlands found that infections among HCWs were more likely to be due to community transmission.^14^ A German study investigated frontline HCW serology on a weekly basis. The low SARS-CoV-2 seroprevalence (1%–2%) was reassuring given concerns that frontline HCWs could have higher incidence during the pandemic.^9^ Consistent with the other emerging studies, we failed to find strong evidence of patient-to-HCW transmission. Instead, we found robust evidence of community transmission, driven by racial and community health disparities.

These results, however, do not mean that occupational risk for HCWs is negligible, as evidenced by the early clusters before universal masking and the modest increase observed among all frontline HCWs when pooled together. HCW health and workforce protection have been focal points of the pandemic response since the initial outbreaks.^1^ Professional guidelines have been published defining strategies to protect HCWs, and PPE use is one of the most important measures.^25,26^ Different levels of PPE have been recommended for different patient-facing scenarios. For example, surgical masks, in addition to standard precautions (gowns, gloves, and handwashing) should be sufficient for HCWs who are not in close contact with diagnosed or suspected COVID-19 patients, although N-95 respirators with standard precautions are recommended for HCWs in close contact with diagnosed and suspected COVID-19 cases.^27^ We observed no infections among our anesthesia teams despite their frequent involvement in intubations of COVID-19 patients. The healthcare system where we conducted the study has also used extended use and reuse of N-95 respirators for all COVID-19–confirmed or –suspected patients since the inception of the pandemic. Thus, our present study suggests that such protective strategies work well for HCWs, which is in agreement with the evidence that PPE offers adequate protection against COVID-19.^10,11,19^

Another novel finding of this study is the racial disparity of COVID-19 incidence in healthcare work environment. Our results show that African-American and Hispanic HCWs are disproportionately affected by COVID-19, even after adjusting for residential area attack rates. These findings of health disparities are in accordance with existing evidence.^13^ In fact, the US CDC has recognized racial minorities as a vulnerable population that needs extra protection during the pandemic. Nationwide statistics as of June 12, 2020, show that African-American and Hispanic populations had a 4–5 times higher COVID-19 hospitalization or death rate than non-Hispanic white populations.^28^ Similar trends have also been demonstrated in the United Kingdom, with the proportion of COVID-19 confirmed cases among racial minorities being double the expected proportion based on the minority proportion of population in the last census.^29^ A recently published review paper discussed potential causes of the racial disparity in COVID-19 pandemic.^29^ Accumulating evidence has shown socioeconomic disadvantages among minorities with regard to their jobs, living conditions, and access to healthcare, among other factors.^29^

The current study has several strengths. First, the occupational variables, demographics, and residential areas were extracted from an established database, and each HCW’s occupational status was classified according to an agreement between the research team, human resources, and infection prevention leadership of the healthcare system. All relevant exposure data were collected before the HCW’s COVID-19 diagnosis, freeing our study from recall bias. Second, the healthcare system has been utilizing consistent policies regarding workers’ screening criteria and PPE use, following professional guidelines, since the initial outbreak. Therefore, the outcome measurement was consistent throughout the study period. Third, the hotline followed each referred HCW and determined whether that HCW needed a second testing, minimizing the possibility of a false negative due to single testing. Fourth, we performed sensitivity analysis for occupational risk factors, and the results remained in agreement with the main analysis, reinforcing our findings. Finally, all adult employees of the healthcare system were included in the study. Nonhealthcare personnel, such as maintenance workers, contractors, and security staff, were also included in the analysis. Therefore, our results may be generalized to larger working populations with similar settings.

This study has some limitations. First, not all workers in the healthcare system were tested, and those who did not undergo hotline triage were assumed to be clinically COVID-19 free. Therefore, we cannot exclude the possibility that these people were asymptomatic or subclinical cases that were not detected. However, the outcome misclassification should not be associated with HCW jobs or demographics, and, therefore, the related information bias is nondifferential. Second, the data on race, sex, and residential area were missing for some contract HCWs. However, we had ∼90% complete information for the analyses, and the missingness was independent of COVID-19 testing; hence, we believe the study results are unbiased. The category assignments of frontline and nonfrontline or patient-facing or non–patient-facing were based on imperfect employment records and may have misclassified some HCWs, but to the extent it occurred, it was likely nondifferential. Finally, we could not evaluate individual compliance with proper handwashing, hygiene, and PPE use.

In conclusion, the current study shows that the major risk factors associated with COVID-19 infection among HCWs are residential area attack rate and race. No significant infection difference between frontline and nonfrontline HCWs were found, neither did high-risk jobs have higher incidence rates. Future studies and public health efforts that can reduce racial and socioeconomic health disparities in the pandemic are warranted.

## Supplementary material

For supplementary material accompanying this paper visit http://dx.doi.org/10.1017/ice.2021.17. click here to view supplementary material
