# Supplementary material for: Sociodemographic risk factors for coronavirus disease 2019 (COVID-19) infection among Massachusetts healthcare workers: A retrospective cohort study
Source: Infect Control Hosp Epidemiol. 2021 Jan 28:1–6. doi: 10.1017/ice.2021.17 (PMC8185417; doi:10.1017/ice.2021.17)
Supplement: Supplementary file 1 [file S0899823X21000179sup001.docx]

**Supplemental Table. COVID-19 infection odds ratio comparing frontline/ non-frontline workers in stratified job groups, adjusting for race and residential community cumulative attack rates, excluding 29 non-index employees involved in infection clusters**

| Job Group Type (n=5148) | Crude Rate | Rate per 100,000 | Adjusted Odds Ratio  (95% CI) ^ab^ | Median Community Rate per 100,000 (95% CI) ^a^ |
| --- | --- | --- | --- | --- |
| Overall |  |  |  |  |
| Frontline/ Patient-facing | 46/1229 | 3742.88 | **1.73 (1.16–2.54)** | 1676.89 (1634.48–1698.80) |
| Non-frontline/ Not Patient-facing | 77/3919 | 1964.79 | 1.0 | 1390.02 (1390.02–1412.36) |
| Registered Nurses (RN) |  |  |  |  |
| Frontline | 12/404 | 2970.30 | 1.23 (0.51–3.00) | 1574.59 (1390.02–1634.48) |
| Non-frontline | 11/557 | 1974.87 | 1.0 | 1181.65 (1152.36–1348.33) |
| Other Nursing (LPN, MA, NA) |  |  |  |  |
| Frontline | 17/178 | 9550.56 | 1.75 (0.87–3.51) | 1698.80 (1698.80–1850.46) |
| Non-frontline | 21/369 | 5691.06 | 1.0 | 1698.80 (1634.48–1698.80) |
| Provider (MD, DO, PA, NP, Resident, Midwife) |  |  |  |  |
| Frontline | 3/140 | 2142.86 | 5.67 (0.80–45.66) | 1152.36 (928.18–1218.63) |
| Non-frontline | 2/763 | 262.12 | 1.0 | 1152.36 (1106.33–1152.36) |
| Clinical Support (Practice manager, Practice Medical Receptionist, etc.) |  |  |  |  |
| Frontline | 1/58 | 1724.14 | 0.63 (0.03–3.60) | 1726.14 (1634.48–2330.25) |
| Non-frontline | 11/366 | 3005.46 | 1.0 | 1698.80 (1698.80–1850.46) |
| Mental Health Providers, Social workers, Psychologists, etc. |  |  |  |  |
| Frontline | 0/5 | 0 | NA | 2014.75 (1699.26–3538.68) ^c^ |
| Non-frontline | 1/208 | 480.77 |  | 1152.36 (1152.36–1634.48) |
| Milieu counselors & Social Workers on In-Patient Psychiatry Units |  |  |  |  |
| Frontline | 1/37 | 2702.70 | 0.70 (0.03–5.83) | 1698.80 (1383.67– 1869.67) |
| Non-frontline | 3/82 | 3658.54 | 1.0 | 1634.48 (1152.36–1698.80) |
| Rehab |  |  |  |  |
| Frontline | 1/14 | 7142.86 | 3.87 (0.16–59.41) | 1698.80 (657.08–2330.25) |
| Non-frontline | 2/87 | 2298.85 | 1.0 | 1412.36 (1152.36–1634.48) |
| Ancillary (Security, Food services, Facilities) |  |  |  |  |
| Frontline | 5/86 | 5813.95 | 1.28 (0.15–8.75) | 1753.47 (1152.36–2330.25) |
| Non-frontline | 5/225 | 2222.22 | 1.0 | 1698.80 (1412.36–1850.46) |
| Radiology Techs - Rad, CT, MRI, Ultrasound, Cardiac |  |  |  |  |
| Patient-facing | 0/49 | 0 | NA | 1390.02 (1181.65–1698.18) |
| Not Patient-facing | 3/70 | 4285.71 |  | 1634.48 (1348.33–1698.80) |
| Pharmacy |  |  |  |  |
| Patient-facing | 1/57 | 1754.39 | 1.23 (0.05–15.97) | 1698.80 (1325.23–1930.59) |
| Not Patient-facing | 2/111 | 1801.80 | 1.0 | 1634.48 (1390.02–1698.80) |
| Lab (Non-MD) |  |  |  |  |
| Patient-facing | 4/114 | 3508.77 | NA | 1698.80 (1412.36–1850.46) |
| Not Patient-facing | 0/1 | 0 |  | NA |
| Interpreter |  |  |  |  |
| Patient-facing | 0/36 | 0 | NA | 1698.80 (1574.59–2330.25) |
| Not Patient-facing | 4/75 | 5333.33 |  | 1634.48 (1383.67–1698.80) |
| Dental Hygiene/ Dental Assistants | 0/12 | 0 | NA | 1693.98 (840.53–2330.25) |
| HR, Finance, IT, Senior Admin, Non-clinical Admin, Medical Records | 7/808 | 866.34 | NA | 1411.34 (1367.75–1634.48) |
| Other |  |  |  |  |
| Frontline | 1/39 | 2564.10 | 1.58 (0.07–13.55) | 1711.27 (1181.65–2136.85) |
| Non-frontline | 5/197 | 2538.07 | 1.0 | 1677.20 (1412.36–1698.80) |

LPN=licensed practical nurse. MA=medical assistant. NA=nursing assistant. MD=medical doctor. DO=doctor of osteopathic medicine. PA=physician assistant. NP=nurse practitioner. CT=computed tomography. MRI=magnetic resonance imaging. HR=human resource. IT=information technology. NA=not available.

^a^ Limited to those residing in New England area.

^b^ Odds ratio adjusted for community rates and race.

^c^ Nonparametric bootstrap confidence intervals were calculated since exact method was not available.
